# 5-azacytidine promotes microspore embryogenesis initiation by decreasing global DNA methylation, but prevents subsequent embryo development in rapeseed and barley

**DOI:** 10.3389/fpls.2015.00472

**Published:** 2015-06-25

**Authors:** María-Teresa Solís, Ahmed-Abdalla El-Tantawy, Vanesa Cano, María C. Risueño, Pilar S. Testillano

**Affiliations:** Pollen Biotechnology of Crop Plants Group, Biological Research Center (CIB) – Spanish National Research Council (CSIC) Madrid, Spain

**Keywords:** microspore culture, epigenetic inhibitors, demethylating agents, totipotency, microspore reprogramming, *Hordeum vulgare*, *Brassica napus*

## Abstract

Microspores are reprogrammed by stress *in vitro* toward embryogenesis. This process is an important tool in breeding to obtain double-haploid plants. DNA methylation is a major epigenetic modification that changes in differentiation and proliferation. We have shown changes in global DNA methylation during microspore reprogramming. 5-Azacytidine (AzaC) cannot be methylated and leads to DNA hypomethylation. AzaC is a useful demethylating agent to study DNA dynamics, with a potential application in microspore embryogenesis. This work analyzes the effects of short and long AzaC treatments on microspore embryogenesis initiation and progression in two species, the dicot *Brassica napus* and the monocot *Hordeum vulgare*. This involved the quantitative analyses of proembryo and embryo production, the quantification of DNA methylation, 5-methyl-deoxy-cytidine (5mdC) immunofluorescence and confocal microscopy, and the analysis of chromatin organization (condensation/decondensation) by light and electron microscopy. Four days of AzaC treatments (2.5 μM) increased embryo induction, response associated with a decrease of DNA methylation, modified 5mdC, and heterochromatin patterns compared to untreated embryos. By contrast, longer AzaC treatments diminished embryo production. Similar effects were found in both species, indicating that DNA demethylation promotes microspore reprogramming, totipotency acquisition, and embryogenesis initiation, while embryo differentiation requires *de novo* DNA methylation and is prevented by AzaC. This suggests a role for DNA methylation in the repression of microspore reprogramming and possibly totipotency acquisition. Results provide new insights into the role of epigenetic modifications in microspore embryogenesis and suggest a potential benefit of inhibitors, such as AzaC, to improve the process efficiency in biotechnology and breeding programs.

## Introduction

Microspore embryogenesis is a fascinating process of cellular reprogramming and totipotency acquisition. In this process, a differentiating cell, the microspore, abandons its gametophytic developmental program in response to the application of a stress treatment *in vitro*, producing a complete embryo capable of germinating and regenerating a haploid or double-haploid mature plant. Microspore embryogenesis has been set up through isolated microspore cultures in several different plant species (Touraev et al., 1997; Massonneau et al., 2005; Forster et al., 2007; Testillano and Risueño, 2009). Microspore embryogenesis is also a powerful biotechnological tool in plant breeding as a method for the rapid production of isogenic lines, generation of new genetic variability and new genotypes, but this technique has had limited efficiency in many crops that are of particular interest (Maluszynski et al., 2003; Germana, 2011). Despite recent advances, there is still little known about the mechanisms that promote reprogramming of differentiating cells and their conversion, in response to stress, into totipotent cells capable of forming an embryo and a plant, without the fusion of the gametes (Grafi et al., 2011).

Stress-induced plant cell reprogramming and acquisition of cellular totipotency involves repression and/or activation of numerous genes associated with the new development program as well as changes in global genome organization (Finnegan et al., 2000). Epigenetic marks are involved in the regulation of global gene expression programs in the genome (Kohler and Villar, 2008). DNA methylation, by DNA methyltransferases, constitutes a prominent epigenetic modification of the chromatin fiber which is associated with gene silencing. This epigenetic mark changes during plant cell differentiation and proliferation processes, and regulates gene expression (Finnegan et al., 2000; Meijón et al., 2010). Recently, work by our group has shown modifications in global DNA methylation that accompanied the change of developmental program of the microspore toward embryogenesis, indicating an epigenetic reprogramming after microspore induction to a totipotent state and embryogenesis initiation. This epigenetic reprogramming involved a global DNA methylation decrease with the activation of cell proliferation, and a subsequent DNA methylation increase with embryo differentiation, in very different plant species, like *Brassica napus* (Solís et al., 2012; Testillano et al., 2013), *Hordeum vulgare* (El-Tantawy et al., 2014), and *Quercus suber* (Rodriguez-Sanz et al., 2014a).

In eukaryotic cells, 5-Azacytidine (AzaC), a known analog of 5-cytosine, inhibits DNA methyl transferase activity leading to genomic DNA hypomethylation (Friedman, 1981). AzaC has been used as a demethylating agent in several different plant systems, leading to a wide range of effects on development depending on the dose, time, and process (Loschiavo et al., 1989; Li et al., 2001; Pedrali-Noy et al., 2001; Santos and Fevereiro, 2002; Yamamoto et al., 2005; Yang et al., 2010; Fraga et al., 2012; Pecinka and Liu, 2014; Teyssier et al., 2014). Treatments with AzaC have also been reported to affect chromosome behavior and structure in root cells (Castilho et al., 1999; Vorontsova et al., 2004). In addition AzaC has been shown to shorten nucleologenesis by early NOR replication, and may possibly lead to early entry of root meristematic cells in the next cell cycle (De-La-Torre et al., 1991; Mergudich et al., 1992). However, there have been no studies with AzaC treatments in isolated microspore cultures and its effects on microspore embryogenesis initiation and progression, in correlation with changes in DNA methylation levels and distribution patterns.

In this work, the effects of AzaC on microspore embryogenesis induction and progression, as well as on global DNA methylation levels, nuclear distribution of methylated DNA and chromatin organization have been analyzed in two plant species, the dicot *B. napus* (rapeseed) and the monocot *H. vulgare* (barley).

## Material and Methods

### Plant Material and Growth Conditions

*Brassica napus* L. cv. Topas (rapeseed) and *Hordeum vulgare* L. cv. Igri (barley) were used as donor plants. Barley seeds were germinated in soil for 1 month at 4°C. After that, they were grown at 12°C with a 12/12 light/dark cycle (10,000–16,000 lx) for 1 month in a plant growth chamber (Sanyo; relative humidity about 70%), and then in a greenhouse under a controlled temperature of 18°C. Rapeseed seeds were sown in soil and plants were grown under controlled conditions at 15/10°C in a 16/8 h light/dark cycle in a plant growth chamber (Sanyo) with 60% relative humidity.

### Microspore Isolation and Culture

Rapeseed microspore culture was performed as previously described (Prem et al., 2012). Selected flower buds containing microspores at the vacuolated stage [the most responsive stage for embryogenesis induction (González-Melendi et al., 1995) were surface-sterilized in 5% commercial bleach for 20 min and then rinsed 6–7 times with sterile distilled water. Ten to fifteen buds were crushed using a cold mortar and pestle in 5 ml of cold NLN-13 medium (Lichter, 1982); Duchefa] containing 13% sucrose (w/v). The suspension was filtered through a 48 μm nylon mesh and the filtrate collected in 15 ml falcon centrifuge tubes. The crushed buds were rinsed with 5 ml NLN-13 to make up the volume to 10 mL and the filtrate was then centrifuged at 185 × *g* for 5 min at 4°C. The pellet was resuspended in 10 mL of cold NLN-13 and centrifuged as mentioned above. This process was repeated three times for washing of the microspores. The final pellet was suspended in the NLN-13, and the cell density was adjusted to 10,000 cells per mL. After isolation, cultures were subjected to 32°C temperature for embryogenesis induction and checked every 2 days under the stereomicroscope till development of globular embryos was observed, around 10 days after culture initiation. Thereafter, cultures were shifted to 25°C on an orbital shaker at 60 rpm (amplitude of rotation: 20 mm) until complete development and maturation of the embryos was observed, around 30 days after culture initiation, as previously described (Prem et al., 2012).

Barley microspore culture was performed as previously described (Rodríguez-Serrano et al., 2012). Spikes containing microspores at the vacuolated stage were collected and surface sterilized by immersion in bleach at 5% for 20 min, followed by 3–4 washes with sterile distilled water. The sterilized spikes were then pre-treated at 4°C for 23–24 days as stress treatment to induce embryogenic development. The isolation and culture of the microspores were performed as previously described (Rodríguez-Serrano et al., 2012) with final density of 1.1 × 10^5^ cell per mL in an appropriate volume of KBP medium (Kumlehn et al., 2006). To isolate the microspores, the spikes were blended in 20 mL of precooled 0.4 M mannitol using a Waring Blender (Eberbach, Ann Arbor, MI, USA) precooled in a refrigerator, and the extract was filtered through a 100 μm nylon mesh (Wilson, Nottingham, UK) into a vessel at 4°C. The microspore suspension collected was transferred into a 50 ml tube and centrifuged at 100 × *g* for 10 min at 4°C. After removing the supernatant, the pellet was resuspended in 8 mL of ice-cold 0.55 M maltose. This volume was distributed between two 15 mL tubes and each aliquot cautiously over layered with 1.5 mL of mannitol solution. After gradient centrifugation at 100 × *g* for 10 min at 4°C, the interphase band consisting of an almost pure population of vacuolated microspores was resuspended in mannitol solution giving a final volume of 20 mL. The pelleted microspores were diluted in an appropriate volume of KBP medium to obtain a cell density of 1.1 × 10^5^ cells per mL. The microspores were incubated at 25°C in the dark. Embryos were observed after around 30 days.

### Treatments of Microspore Cultures with AzaC

The demethylating agent 5-AzaC (Sigma) was added to the culture plates at the culture initiation from a freshly prepared concentrated solution of 500 μM in culture media, after filtering with a sterile Ministart filter (Sartorius Biotech). In a first experiment, this solution was added to rapeseed microspore cultures at three different concentrations, 2.5, 5, and 10 μM, keeping parallel plates without the drug as control. The rest of treatments were performed at the selected concentration of 2.5 μM.

Short AzaC treatments were performed from culture initiation during 4 days, time of the proembryo formation stage in both *in vitro* microspore cultures, rapeseed (Prem et al., 2012) and barley (Rodríguez-Serrano et al., 2012).

Long AzaC treatments were carried out from culture initiation until the stage of embryo formation (cotyledonar embryos in rapeseed and coleoptilar embryos in barley), during 30 days in both systems (Prem et al., 2012; Rodríguez-Serrano et al., 2012).

Quantification of the number of three types of structures, “proembryos,” “developing embryos,” and “embryos” was performed at defined time points of the cultures. Quantifications were carried out using stereomicroscope micrographs randomly obtained from control and AzaC-treated microspore culture plates. “Proembryos” were rounded multicellular structures, still surrounded by the exine, which displayed higher size and density than microspores. “Developing embryos” were structures formed after the exine breakdown and much larger than proembryos; these term “developing embryos” included embryos at different developmental stages of the two pathways (monocot and dicot species). Mean percentages of “proembryos” and “developing embryos,” and total number of “embryos” (fully developed) per Petri dish were calculated from random samples of two independent experiments and 10–15 different culture plates per each *in vitro* system. A total of 100–140 micrographs and 1000–1800 embryo structures were evaluated for each culture time point, each treatment, and each plant species. The results were shown in histograms in which columns represented mean values and bars represented SEM. Significant differences between non-treated (control) cultures and AzaC-treated cultures were tested by Student’s *t*-test at *P* ≤ 0.05.

### Cell Death Detection and Quantification

To determine changes in viability of cells, detection of dead cells in microspore cultures was performed by Evans blue staining (Rodríguez-Serrano et al., 2012) in control and AzaC-treated cultures. Culture samples were incubated with a 0.25% (w/v) aqueous solution of Evans Blue for 30 min and observed with a light microscope under bright field. The number of dead (stained by Evans Blue) and live (unstained by Evans Blue) cells were quantified on random micrographs from two replicas (Evans blue-stained preparations) and three independent samples of each culture treatment; mean percentages of dead cells were calculated. A total of 150–200 micrographs and 2000–2500 structures were evaluated per culture treatment. The results were shown in histograms in which columns represented mean values and bars represented SEM. Significant differences in the percentage of dead cells between non-treated (control) cultures and AzaC-treated cultures at different concentrations were tested by Student’s *t*-test at *P* ≤ 0.05.

### Quantification of Global DNA Methylation

Genomic DNA was extracted from samples of microspore cultures of rapeseed and barley at the stage of proembryo formation (4 days), in non-treated conditions and after short treatments with 2.5 μM AzaC. The DNA extraction was performed using a plant genomic DNA extraction kit (DNeasy Plant Mini, Qiagen) as previously described (Solís et al., 2014). A MethylFlash Methylated DNA Quantification Kit (Colorimetric; Epigentek, Farmingdale, NY, USA) was used for the quantification of the global DNA methylation according to the manufacturer’s instruction, using 200 ng of genomic DNA (Testillano et al., 2013) collected from various culture plates of each sample (for barley: 20–25 plates of 50 mm diameter and 1.5 mL of culture medium each; for rapeseed: 8–10 plates of 90 mm diameter and 15 mL of culture medium each). Three biological (independent culture experiments) and two analytical (DNA methylation colorimetric assays) replicates per sample were taken and mean percentages of 5-methyl-deoxy-cytidine (5mdC) of total DNA were calculated. The results were shown in histograms in which columns represented mean values and bars represented SEM. Significant differences between non-treated (control) cultures and AzaC-treated cultures were tested by Student’s *t*-test at *P* ≤ 0.05.

### Fixation and Processing for Light Microscopy Analysis

Samples from different culture times were collected and fixed overnight at 4°C with 4% paraformaldehyde in phosphate buffered saline (PBS) pH 7.3. Culture samples of the first stages contained isolated microspores and small multicellular proembryos, they were previously embedded in gelatine. After fixation, samples were washed in PBS, dehydrated in an acetone series, embedded in Historesin Plus at 4°C and sectioned at 2 μm thickness using an ultramicrotome (Ultracut E Reichert). Some semithin resin sections were stained with 1% toluidine blue, for structural analysis, mounted with Eukitt, and observed under bright field microscopy. Other sections were stained with 1 mg mL^-1^ DAPI (4′,6-diamidino-2-phenylindole), specific staining for DNA, for 10 min, for observation of the nuclei under UV excitation and epifluorescence microscopy.

### 5mdC Immunofluorescence and Confocal Microscopy

Immunolocalization of 5mdC was performed as previously described (Solís et al., 2012; Testillano et al., 2013). Historesin semithin sections were mounted on 3-aminopropyltriethoxysilane- coated slides, denatured with 2N HCl for 45 min, washed in PBS and treated with 5% bovine serum albumin (BSA) in PBS for 10 min, incubated with anti-5mdC mouse antibody (Eurogentec) diluted 1/50 in 1% BSA and Alexa-Fluor-488 anti-mouse IgG antibody (Molecular Probes) diluted 1/25. As negative controls, either DNA denaturation step or first antibody was omitted. Sections were counterstained with 1 mg mL^-1^ DAPI (4′,6-diamidino-2-phenylindole) for 10 min and analyzed by confocal laser microscopy (TCS-SP5, Leica). Images of maximum projections were obtained with software running in conjunction with the confocal microscope (Leica software LCS version 2.5). Confocal microscopy analysis was performed using the same laser excitation and sample emission capture settings in all immunofluorescence preparations of each species, rapeseed or barley, allowing an accurate comparison between signals of control and AzaC-treated cells.

### Electron Microscopy and Ultrastructural Analysis

Samples to be observed for transmission electron microscopy (TEM) were processed and embedded in Epon 812 or K4M Lowicryl resin, as previously described (Testillano et al., 2005; Solís et al., 2014). Samples to be embedded in Epon resin were fixed in Karnovsky fixative (4% formaldehyde + 5% glutaraldehyde in 0.025M cacodylate buffer, pH 6.7), dehydrated in a methanol series for 3 days and slowly embedded in Epon resin for 2 days. Epon blocks were polymerized at 60°C for 2 days. Samples to be embedded in K4M Lowicryl were fixed in 4% formaldehyde in PBS at 4°C, overnight, dehydrated in a methanol series by Progressive Lowering of Temperature (PLT) and embedded in K4M Lowicryl at -30°C, in an Automatic Freeze-Substitution unit (AFS, Leica, Vienna). 80 nm thick ultrathin sections were collected on 75 mesh copper grids, counterstained with uranyl acetate and lead citrate and observed in a JEOL 1010 TEM operating at 80 kV.

### 5mdC Immunogold Labeling for Electron Microscopy

Immunogold labeling for 5mdC ultrastructural localization was performed as previously described (Solís et al., 2014). Lowicryl ultrathin sections were obtained and collected on 200 mesh nickel grids with a carbon-coated Formvar supporting film. Ultrathin sections were floated on drops of distilled water, denaturated with 2N HCl for 45 min and washed in PBS before incubation in 5% BSA. For immunogold labeling, they were incubated with anti-5mdC antibody (diluted 1:50) for 1 h at room temperature. After washing with PBS, the sections were incubated with anti-mouse secondary antibody conjugated to 10 nm gold particles (BioCell) diluted 1:25 in PBS for 45 min. Then, the grids were washed in PBS, rinsed in distilled water and air-dried. Negative controls were performed by omitting either the DNA denaturation step or the first antibody. Finally, the grids were counterstained with 5% uranyl acetate and 1% lead citrate, and observed with a JEOL 1010 microscope operating at 80 kV.

## Results

### Effects of Short AzaC Treatments on Microspore Embryogenesis Initiation

**FIGURE 1:**
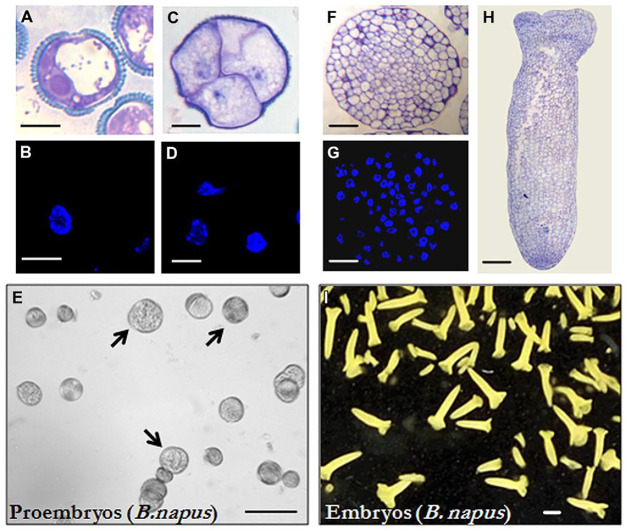
**Microspore embryogenesis in *Brassica napus*. (A,B)** Vacuolated microspores at the beginning of the culture. **(A)** was reproduced from Figure 1A of Rodriguez-Sanz et al. (2014b) (copyright^©^ 2014 Karger Publishers, Basel, Switzerland). **(C,D)** Proembryos formed by four cells, still surrounded by the exine (the microspore wall). **(E)** *In vitro* culture at the proembryo formation stage (4 days), proembryos are pointed by arrows. **(F,G)** Globular embryos. **(H)** Torpedo embryo. **(I)** *In vitro* culture at the embryo production stage (30 days), most embryos show the typical morphology of cotyledonary embryos of the dicot embryogenesis pathway, some embryos at earlier developmental stages (heart and torpedo embryos) are also present. **(A,C,F,H)** Micrographs of toluidine blue-stained sections for general structure visualization. **(B,D,G)** DAPI staining for nuclei visualization (blue). **(E,I)** General views of cultures observed under the stereomicroscope. Bars represent, in **(A–D)** 10 μm, in **(E)** 250 μm, in **(F,G)** 50 μm, in **(H)** 100 μm, in **(I)** 1mm.

**FIGURE 2:**
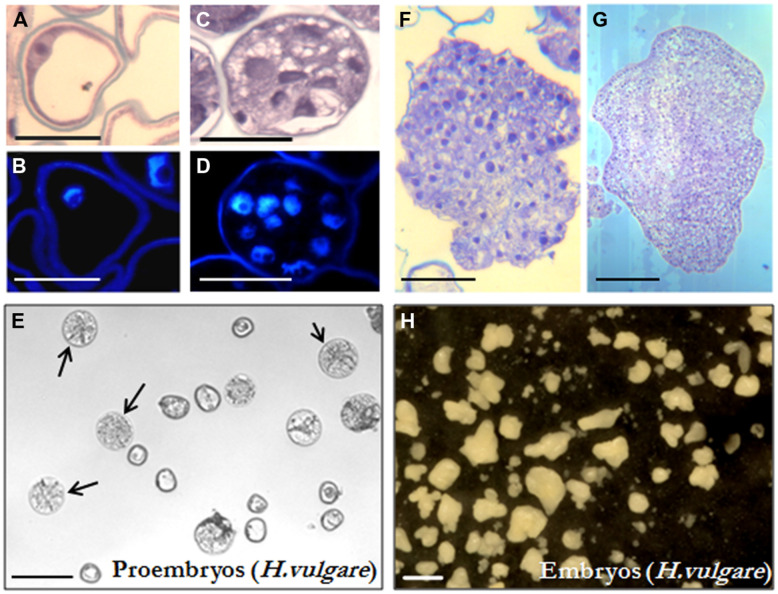
**Microspore embryogenesis in *Hordeum vulgare*. (A,B)** Vacuolated microspores at the beginning of the culture. **(C,D)** Proembryos formed by several cells, still surrounded by the exine (the microspore wall). **(E)** *In vitro* culture at the proembryo formation stage (4 days), proembryos are pointed by arrows. **(F,G)** Early and late transitional embryos. **(H)** *In vitro* culture at the embryo production stage (30 days), embryos show the typical morphology of coleoptilar embryos of the monocot embryogenesis pathway, some embryos at earlier developmental stages (globular, early, and late transitional and scutellar embryos) are also present. **(A,C,F,G)** Micrographs of toluidine blue-stained sections for general structure visualization. **(B,D)** DAPI staining for nuclei visualization (blue). **(E,H)** General views of cultures observed under the stereomicroscope. Bars represent, in **(A,B)** 20 μm, in **(C,D)** 50 μm, in **(E)** 250 μm, in **(F,G)** 100 μm, in **(H)** 1 mm.

Isolated microspore *in vitro* cultures were set up and embryogenesis induction performed, both according to previously described protocols in *B. napus* (Prem et al., 2012) and *H. vulgare* (Rodríguez-Serrano et al., 2012), as described in the “Materials and Methods” section. Vacuolated microspores (**Figures 1A,B** and **2A,B**), the most responsive developmental stage for embryogenesis induction in both monocot and dicot species (González-Melendi et al., 1995; Testillano et al., 2002, 2005), were subjected to the corresponding inductive stress treatment for each system, i.e., 32°C for *B. napus* and 4°C for *H. vulgare*. Four days after induction and culture initiation, responsive microspores that initiated the embryogenesis pathway had divided and produced multicellular structures still surrounded by the exine, the so-called microspore-derived “proembryos” (**Figures 1C,D** and **2C,D**). These proembryos (arrows in **Figures 1E** and **2E**) were clearly distinguished from the non-responsive microspores present in the culture, they were rounded structures displaying higher size and density than microspores, in both *in vitro* systems, rapeseed and barley. Over the following days in culture, microspore embryogenesis progressed; the exine broke down, and embryos developed following a pathway similar to the zygotic embryogenesis in monocot and dicot species. In the case of rapeseed, globular (**Figures 1F,G**), heart, torpedo (**Figure 1H**), and cotyledonary embryos (**Figure 1I**) were formed (Prem et al., 2012), while in barley microspore cultures globular, transitional, scutellar, and coleoptilar monocot embryos (**Figures 2F–H**) were developed (Rodríguez-Serrano et al., 2012).

**FIGURE 3:**
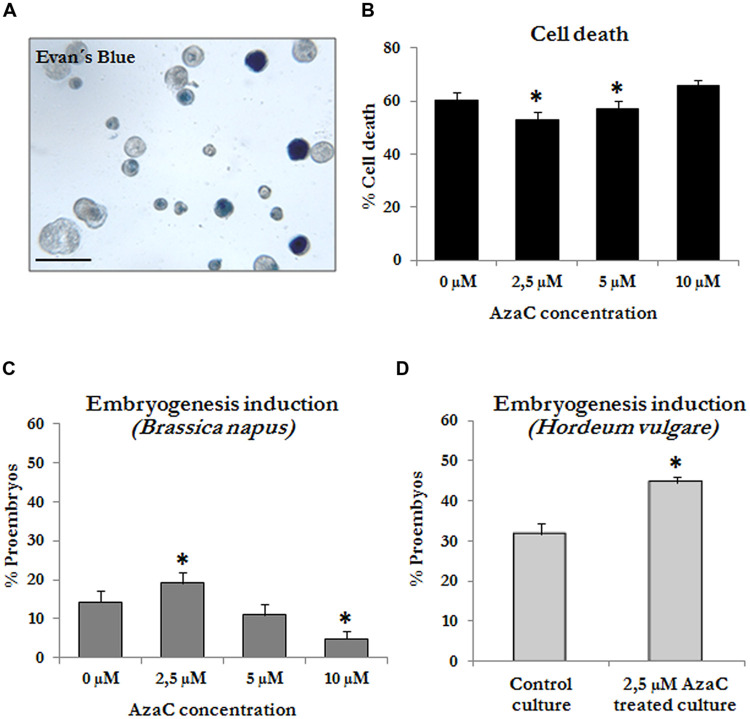
**Effects of short Azacytidine (AzaC) treatment in microspore cultures on cell death and embryogenesis induction. (A)** Evan’s blue staining to detect dead cells in microspore embryogenesis cultures of *B. napus* at the proembryo formation stage. The staining solution only enters into dead cells, which appeared blue. **(B,C)** Quantification of the percentage of dead cells **(B)** and proembryos **(C)** in microspore cultures of *B. napus* at the proembryo formation stage, after short treatment (4 days) with AzaC at the concentrations of 0 μM (control), 2.5, 5, and 10 μM. **(D)** Quantification of the percentage of proembryos in microspore cultures of *H. vulgare*, after short treatments (4 days) with AzaC at the concentrations of 0 μM (control) and 2.5 μM. Bar in **(A)** represents 100 μm. In histograms **(B–D)**, columns represent mean values and bars represent SEM; asterisks indicate significant differences with the non-treated/control culture sample (Student’s *t*-test at *P* ≤ 0.05).

Firstly, different concentrations of AzaC, 2.5, 5.0, and 10 μM, were tested during short treatments (4 days) on rapeseed microspore cultures, and their effects on both, cell death, and microspore embryogenesis initiation efficiency (proembryo formation) were evaluated. The percentage of dead cells, identified by positive Evans blue staining (**Figure 3A**), present in cultures at the proembryo formation stage (**Figure 1E**) were quantified. Results showed a high level of dead cells in control cultures at the proembryo formation stage. Cell death may be contributed by both the isolation and *in vitro* culture procedures and by the application of the stress treatment on non-responsive mcirospores (**Figure 3B**). Microspore cultures treated with 2.5 and 5 μM AzaC showed a small but statistically significant reduction in cell death, in comparison with control cultures (**Figure 3B**).

Quantifications of proembryos at the same culture time point showed significant higher proportion of these multicellular structures upon 2.5 μM AzaC treatment compared to control cultures (**Figure 3C**). By contrast, higher AzaC concentrations (5 and 10 μM) reduced the proportion of proembryos. Therefore, the concentration of 2.5 μM was selected for the subsequent AzaC treatments in microspore cultures.

Short AzaC treatments were also applied to barley microspore cultures, at the concentration of 2.5 μM, by adding the drug to the culture medium from the beginning of the culture until the proembryo formation stage (4 days). The quantification of the proembryos formed in untreated and AzaC-treated microspore cultures of barley revealed that short AzaC treatments also produced a significantly higher proportion of proembryos in comparison with non-treated cultures (**Figure 3D**) in barley, like in rapeseed.

### Effects of Short AzaC Treatments on Global DNA Methylation Levels and Distribution Patterns of Methylated DNA

**FIGURE 4:**
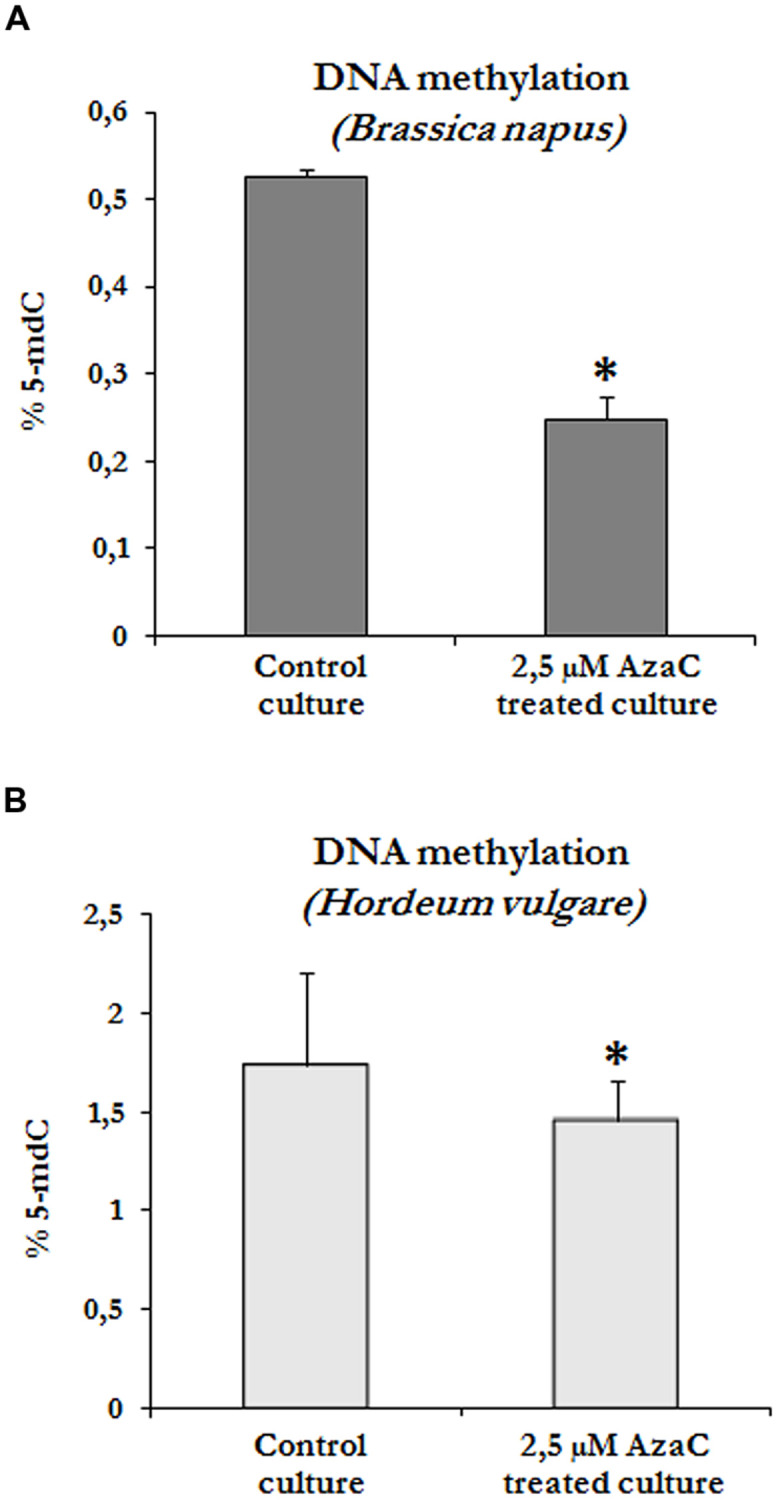
**Effects of short AzaC treatment in microspore embryogenesis cultures on global DNA methylation levels.** Quantification of global DNA methylation levels in control and 2.5 μM AzaC-treated cultures of *B. napus* **(A)** and *H. vulgare* **(B)**, at the proembryo formation stage. Columns represent mean values and bars represent SEM of 5-methyl-deoxy-cytidine (5mdC) percentage of total DNA. Asterisks indicate significant differences with the non-treated/control cultures (Student’s *t*-test at *P* ≤ 0.05).

To evaluate whether the presence of AzaC at a concentration of 2.5 μM affected the DNA methylation of cells in microspore embryogenesis cultures, global DNA methylation levels were quantified in control and treated cultures of rapeseed and barley after short AzaC treatments (4 days), from the beginning of the culture until the proembryo formation stage (**Figures 1E** and **2E**). Results showed significant decreases in global DNA methylation after the AzaC treatments in both plant species (**Figure 4**). In *B. napus* microspore cultures treated by AzaC, DNA methylation levels reached only half of that in control cultures (**Figure 4A**). In barley microspore cultures, the level of methylated DNA also diminished after AzaC treatment (**Figure 4B**), but to a lesser extent than in rapeseed cells.

**FIGURE 5:**
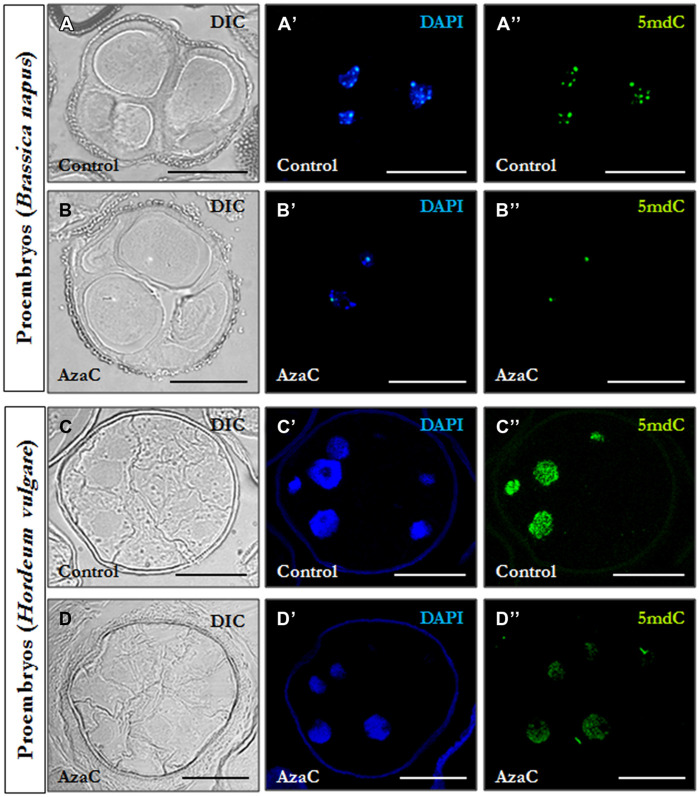
**Distribution patterns of methylated DNA in microspore proembryos under control conditions and short AzaC treatment.** 5mdC immunofluorescence and confocal laser scanning microscopy analysis in *B. napus* **(A,B)** and *H. vulgare* **(C,D)** microspore proembryos of control **(A,C)** and 2.5 μM AzaC-treated **(B,D)** cultures. **(A–D)** Nomarsky’s differential interference contrast (DIC) images of the proembryo structure. **(A′–D′)** DAPI staining of nuclei (blue). **(A^′′^–D^′′^)** 5mdC immunofluorescence (green). The same structures are visualized under different microscopy modes in **(A–A^′′^, B–B^′′^, C–C^′′^, and D–D^′′^)**. The exine showed unspecific autofluorescence under UV excitation in some DAPI images **(C′,D′)**. Bars represent 20 μm.

Immunofluorescence assays with 5mdC antibodies and confocal laser scanning microscopy analysis were performed to analyze the effects of short AzaC treatments on the nuclear localization pattern of methylated DNA. Immunofluorescence images of treated samples were obtained in the confocal microscope under the same excitation intensity and emission capture settings than the non-treated samples, allowing an accurate comparison between signals. In non-treated cultures of rapeseed, microspore-derived proembryos were formed by several cells with a central rounded nucleus each, separated by straight cell walls and surrounded by the microspore wall, the exine (**Figure 5A**). The 5mdC immunofluorescence signal was concentrated in 4-to-6 conspicuous foci preferentially at the nuclear periphery and associated with heterochromatin foci (condensed chromatin masses), which were also revealed by the DAPI specific staining of DNA (**Figures 5A’,A”**). In microspore cultures treated with 2.5 μM AzaC, proembryos exhibited a cellular organization similar to that in control cultures (**Figure 5B**). Nevertheless, the immunofluorescence assays showed a different nuclear pattern of 5mdC distribution with very low or no 5mdC signal concentrated in 1-to-2 small foci per nucleus (**Figures 5B’,B”**).

Barley microspore-derived proembryos, still surrounded by the exine, displayed numerous small cells with large nuclei and wavy cell walls (**Figure 5C**), which is the typical organization of microspore proembryos in monocot species like barley (Ramírez et al., 2001) and maize (Testillano et al., 2002). No significant differences on the structural organization of proembryos were observed in AzaC-treated cultures (**Figure 5D**). In control cultures, the 5mdC immunofluorescence signal was intense, covering the whole nucleus (**Figures 5C’,C”**) which also exhibited an intense fluorescence intensity by DAPI (**Figure 5C**’). In proembryos developed in the presence of AzaC, the 5mdC immunofluorescence signal was less intense and was distributed over the entire nucleus (**Figures 5D’,D”**). Negative controls avoiding either the DNA denaturation step or the first antibody did not provide any labeling in the nucleus or any subcellular compartment, in any of the plant species analyzed.

### Effects of Short AzaC Treatments on Chromatin Condensation Patterns

**FIGURE 6:**
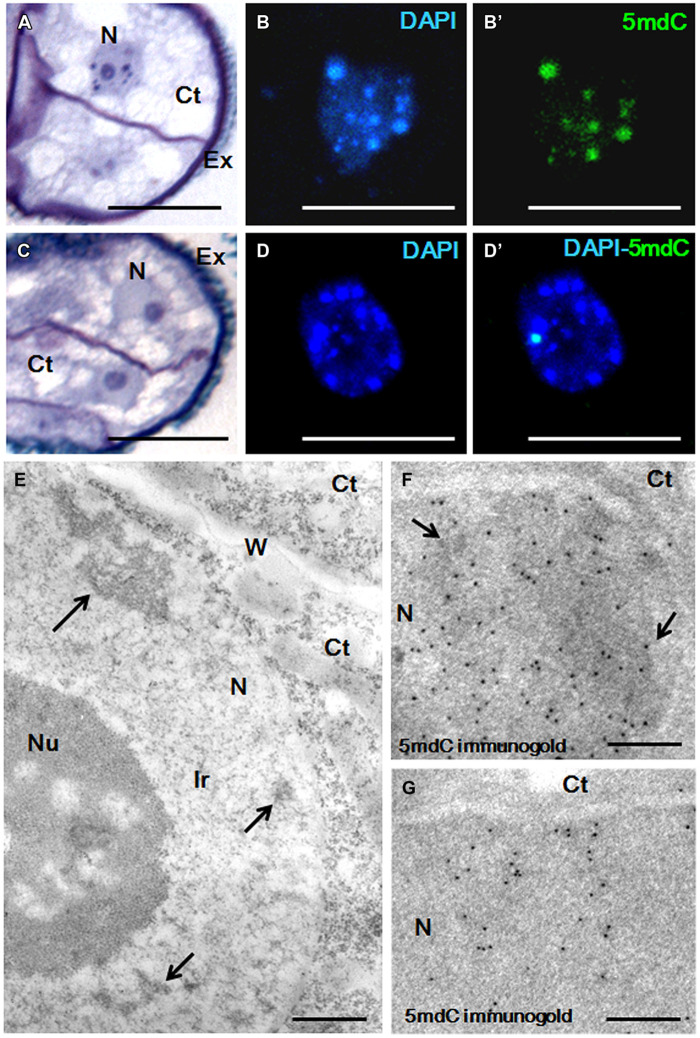
**Chromatin condensation patterns and methylated DNA distribution in microspore proembryos of *B. napus*. (A–D)** High magnification light microscopy images of microspore proembryo nuclei in control **(A,B,B**’) and 2.5 μM AzaC-treated **(C,D,D**’) cultures, observed after toluidine blue staining **(A,C)**, DAPI staining **(B,D)** and 5mdC immunofluorescence **(B’,D’)** by confocal laser scanning microscopy. The same nuclei are visualized under different microscopy modes in **(B,B**’), and in **(D,D**’). **(E–G)** Transmission electron microscopy (TEM) micrographs of nuclear regions of proembryos of control cultures. **(E)** Ultrastructural organization of the nucleus that shows some condensed chromatin masses (arrows), an extensive interchromatin region (Ir) and a large nucleolus (Nu). **(F,G)** 5mdC immunogold labeling over nuclear regions of proembryo cells; large heterochromatin masses (arrows in **F**) are labeled by numerous gold particles, and nuclear regions with small condensed chromatin masses of different sizes show lower labeling **(G)**. No gold particles are found on nucleolus and cytoplasms (Ct). Ex, exine; W, cell wall separating proembryo cells. Bars represent in **(A–D)** 10 μm, in **(E)** 0.5 μm, in **(F)**, **(G)** 0.2 μm.

**FIGURE 7:**
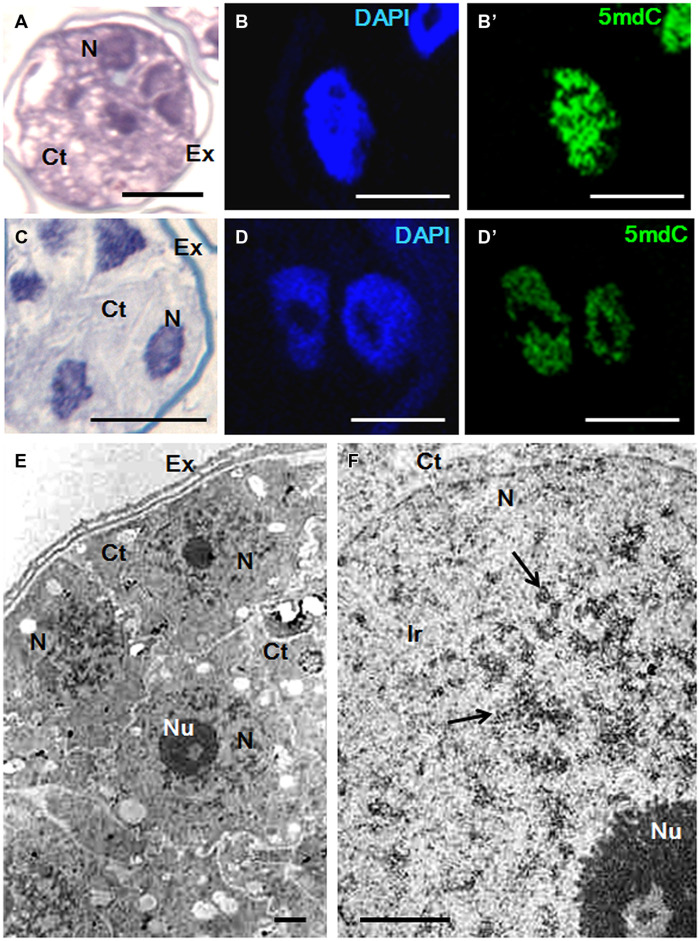
**Chromatin condensation patterns and methylated DNA distribution in microspore proembryos of *H. vulgare*. (A–D)** High magnification light microscopy images of microspore proembryo nuclei in control **(A,B,B′)** and 2.5 μM AzaC-treated **(C,D,D′)** cultures observed after toluidine blue staining **(A,C)**, DAPI staining **(B,D)** and 5mdC immunofluorescence **(B′,D′)** by confocal laser scanning microscopy. The same nuclei are visualized under different microscopy modes in **(B,B′)**, and **(D,D′)**. **(E,F)** TEM micrographs of proembryos of control cultures. **(E)** Panoramic view of a proembryo surrounded by the microspore wall, the exine (Ex) showing several cells with one large nucleus (N) per cell and dense cytoplasms (Ct). **(F)** Detail of a nuclear region at high magnification; condensed chromatin masses (arrows) appear dense to electrons and forming numerous patches of different sizes, frequently connected by chromatin threads. Ir, interchromatin region; Nu, Nucleolus. Bars represent in **(A,C)**: 20 μm, in **(B,B′,D,D′)** 10 μm, in **(E,F)** 1 μm.

Changes in the chromatin condensation degree/pattern of proembryo cells after short AzaC treatments were analyzed in relation to the distribution of methylated DNA, by light and electron microscopy (**Figures 6** and **7**). After toluidine blue staining, nuclei of rapeseed proembryos appeared very clear, with several dark regions, mainly located at the nuclear periphery, as revealed by light microscopy (**Figure 6A**). High magnification fluorescence images of DAPI-stained samples showed a discrete number of brightly stained heterochromatin foci of variable size dispersed in euchromatin, which exhibited lower fluorescence (**Figure 6B**). The 5mdC immunofluorescence signal was intense in the heterochromatin regions while not excluded from euchromatin, which showed a faint 5mdC immunofluorescence signal throughout the nucleus (**Figure 6B**’). After the treatment with AzaC, proembryo nuclei showed a homogeneous chromatin distribution in both toluidine blue (**Figure 6C**) and DAPI (**Figure 6D**) staining with no or little apparent heterochromatin foci. Concomitantly, the 5mdC immunofluorescence signal was very low and occasionally accumulated at one or two bright nuclear foci (**Figure 6D**’).

Transmission electron microscopy analysis revealed the chromatin ultrastructural organization of rapeseed proembryo nuclei, which exhibited a very low condensed chromatin pattern (**Figure 6E**) with a few isolated and electron dense condensed chromatin masses (arrows in **Figure 6E**), which occupied a low fraction of the nuclear volume and were mainly located at the nuclear periphery. These condensed chromatin masses most likely corresponded to the dark spots of heterochromatin observed at light microscopy, in toluidine blue-stained preparations. A large fraction of the nuclear volume was occupied by a wide interchromatin region (Ir) that displayed abundant fibrillo-granular ribonucleoprotein structures (RNPs), which are typical of this nuclear domain (Testillano et al., 2000, 2005; Seguí-Simarro et al., 2011). Together with the RNPs, decondensed chromatin fibers of different thicknesses (euchromatin) were localized (**Figure 6E**). 5mdC immunogold labeling revealed the ultrastructural distribution of methylated DNA; numerous gold particles were found decorating the large condensed chromatin masses, while no labeling was observed in decondensed chromatin (**Figure 6F**). Much less 5mdC immunogold labeling was found in the rest of the nucleus, with only a few gold particles observed as clusters on the very small masses of condensed chromatin, and as isolated particles (**Figure 6G**). The results of the 5mdC immunogold labeling correlated with the distribution of the 5mdC immunofluorescence on the heterochromatin. Negative controls avoiding either the denaturation step or the first antibody did not provide gold labeling on the nucleus or any subcellular compartment.

In barley proembryos, a completely different chromatin organization was found. In control cultures, nuclei of barley proembryos appeared densely stained by toluidine blue (**Figure 7A**); this staining revealed a dense chromatin pattern distributed throughout the entire nuclear area. By contrast, barley proembryos of AzaC-treated cultures showed lower toluidine blue staining density in their nuclei (**Figure 7C**), indicating a less condensed chromatin pattern than in control samples. DAPI staining provided an intense fluorescence to proembryo nuclei of non-treated cultures (**Figure 7B**) while nuclei of AzaC-treated proembryos showed less intense DAPI fluorescence (**Figure 7C**), revealing a less condensed chromatin pattern in treated nuclei. In control proembryos, the signal of 5mdC immunofluorescence was intense and distributed in a reticular pattern (**Figure 7B**’). AzaC-treated nuclei showed a less intense distribution pattern of 5mdC immunofluorescence (**Figure 7D**’), when observed under the confocal microscope with the same excitation and capture settings as those used in non-treated nuclei. These observations suggested a decrease in the degree of chromatin condensation in AzaC-treated nuclei. Nucleoli appeared as non-stained (dark) rounded regions inside the nucleus in both DAPI and immunofluorescence images (**Figures 7B,B’,D,D’**).

Ultrastructural analysis by TEM showed the pattern of chromatin condensation in barley proembryo nuclei (**Figure 7E**). High magnification electron micrographs showed heterochromatin patches distributed throughout the whole nucleus, connected by chromatin threads of different thicknesses (**Figure 7F**). In this species, the abundant condensed chromatin masses (heterochromatin) occupied a significant proportion of the nucleus in comparison with the euchromatin (decondensed chromatin). The Ir that typically contained fibrillo-granular RNPs was less abundant in barley than in rapeseed proembryo nuclei (compare **Figures 6E** and **7F**). The ultrastructural analysis of the condensed chromatin pattern of barley proembryo nuclei revealed that the distribution pattern of the heterochromatin corresponded to that of the methylated DNA revealed by 5mdC immunolocalization assays.

### Effects of Long AzaC Treatments on Microspore-Derived Embryo Development

**FIGURE 8:**
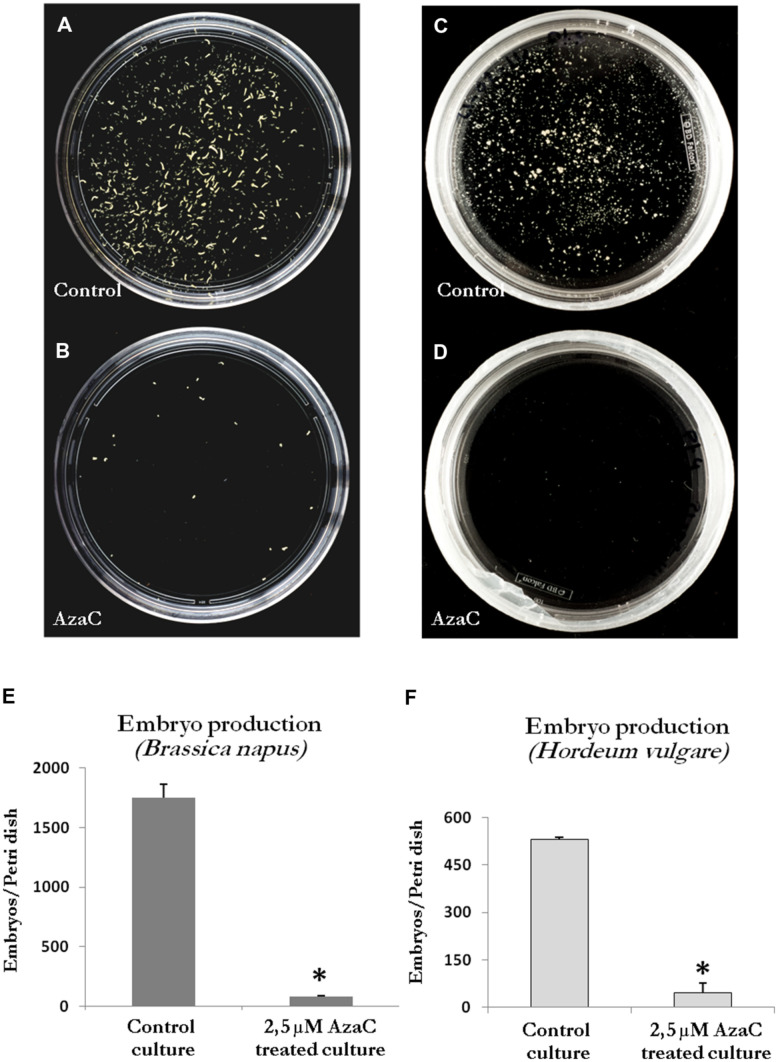
**Effects of long AzaC treatment on embryo production yield. (A–D)** Plates showing the microspore-derived embryos produced in control **(A,C)** and 2.5 μM AzaC-treated **(B,D)** cultures of *B. napus* **(A,B)** and *H. vulgare* **(C,D)**, after 30 days. **(E,F)** Quantification of the embryo production in control and 2.5 μM AzaC-treated cultures of *B. napus* **(E)** and *H. vulgare* **(F)**. In histograms **(E,F)**, columns represent mean values and bars represent SEM of the total number of embryos per Petri dish. Asterisks indicate significant differences with the non-treated/control culture sample (Student’s *t*-test at *P* ≤ 0.05).

Long treatments with AzaC (30 days from culture initiation, the period in which most embryos finished their development) were carried out to evaluate the effects of the drug on embryo production, in the two stress-induced microspore embryogenesis systems, rapeseed and barley. Parallel cultures were performed in the presence and absence of the drug and the production of embryos were analyzed in the two *in vitro* systems at the embryo production stage, after 30 days of culture initiation. The embryos found were late torpedo and cotyledonary embryos in rapeseed (**Figure 1I**) and late scutellar and coleoptilar embryos in barley (**Figure 2H**). The results showed a very marked reduction of embryo production in 2.5 μM AzaC-treated cultures in which only very few embryos were found in both species, in contrast with control cultures which exhibited numerous embryos (**Figures 8A–D**). The quantification of embryos in control and AzaC-treated cultures demonstrated a large decrease in the level of embryo production induced by the drug, in both systems (**Figures 8E,F**).

**FIGURE 9:**
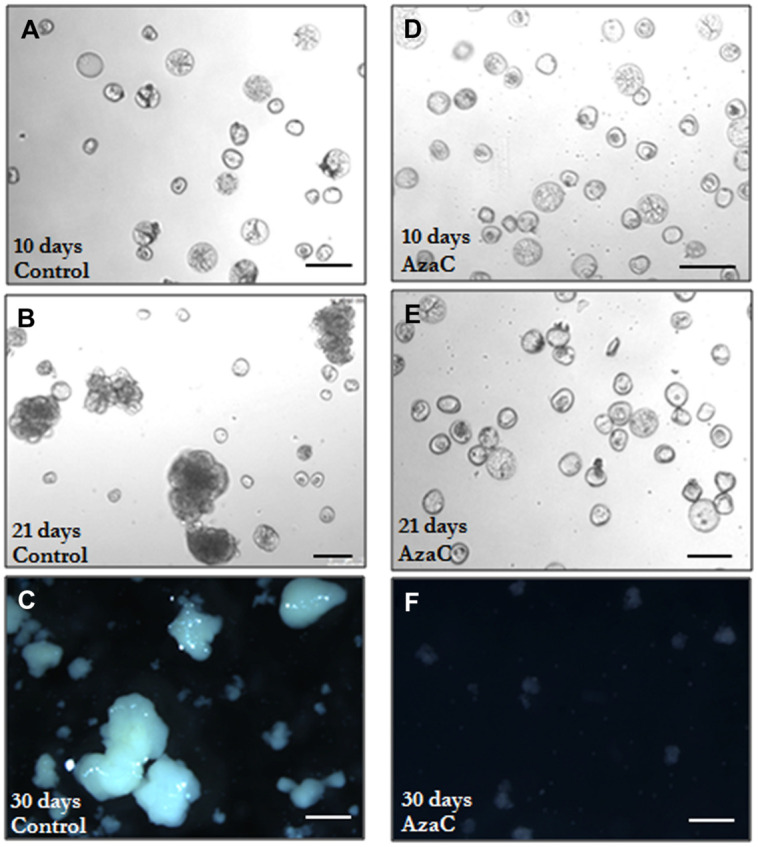
**Progression of microspore embryogenesis in control and AzaC-treated cultures of barley.** Micrographs of microspore cultures observed at different time points. **(A–C)** Control cultures. **(D–F)** 2.5 μM AzaC-treated cultures. **(A,D)** 10 day-old cultures showing typical rounded proembryos surrounded by the exine, clearly distinguished by their size and density (higher than those of microspores), together with non-responsive and dead microspores; in AzaC-treated cultures **(D)** a higher proportion of proembryos than in control cultures is observed. **(B,E)** 21 day-old cultures; control cultures **(B)** show developing embryos of different sizes which were formed after the breakdown of the exine, they exhibit much larger size and more density than the proembryos and microspores still present in the culture. AzaC-treated cultures **(E)** do not progress and contain mostly proembryos. **(C,F)** 30 day-old cultures; in control cultures **(C)** embryos at advanced developmental stages (transitional and coleoptilar embryos) are observed, whereas no embryos are found in AzaC-treated cultures **(F)** at the same time point.

**FIGURE 10:**
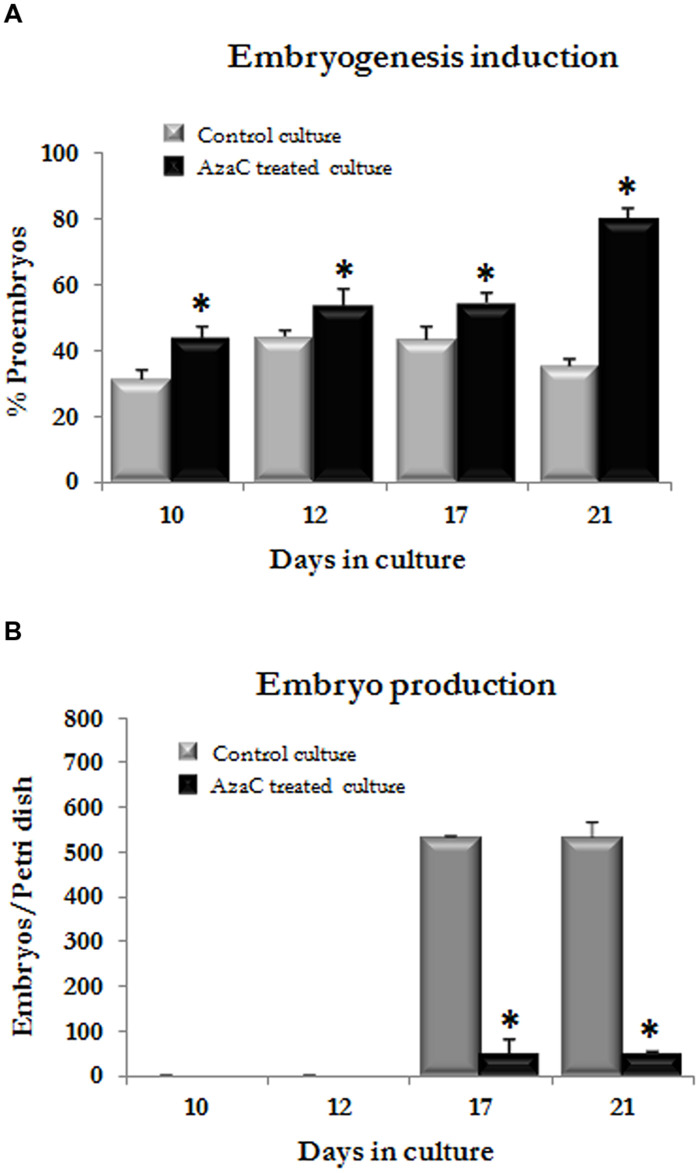
**Effects of long AzaC treatment on microspore embryogenesis progression in barley.** Quantification of the percentage of proembryos **(A)** and developing embryos **(B)** observed at different time intervals (10, 12, 17, and 21 days) during microspore embryogenesis progression in control (gray columns) and 2.5 μM AzaC-treated (black columns) cultures of barley. Columns represent mean values and bars represent SEM. Asterisks indicate significant differences with the non-treated/control culture sample at each time point, days in culture (Student’s *t*-test at *P* ≤ 0.05).

To assess the effects of AzaC on the progression of microspore embryogenesis after the proembryo stage, in barley microspore cultures, treated and non-treated-cultures were monitored under the microscope every few days until the stage in which the first coleoptilar embryos were observed, at 21 days. The number of proembryos (still surrounded by the exine) and the number of developing embryos (embryos at different developmental stages, formed after the exine breakdown) found in control and AzaC-treated cultures were quantified at each time interval (**Figures 9** and **10**).

In control cultures, responsive microspores divided during the first days of culture and produced proembryos which reached a proportion of one third by 10 days (**Figures 9A** and **10A**). Later, the number of proembryos slightly increased until day 12, remained relatively stable for several more days and progressively decreased until day 21 (**Figures 9B** and **10A**). However, in AzaC-treated cultures, the proportion of proembryos at day 10 was significantly higher than in control cultures (**Figures 9D** and **10A**). During the following days, the number of proembryos in AzaC-treated cultures progressively increased, until day 21 (**Figures 9E** and **10A**). The proembryos formed during long AzaC treatments showed similar morphology and size to the proembryos formed in non-treated cultures at early stages (**Figures 9A,D,E**), and no aberrant embryo morphologies were observed during long AzaC treatments. These observations suggested that, in long AzaC treatments, the proembryos that were formed in the presence of the drug during the first days of culture, later stopped developing.

In non-treated cultures, after the exine breakdown embryogenesis progressed and further cell proliferation and differentiation events, that occurred asynchronously, lead to the formation of embryos with various sizes and shapes, the so-called “developing embryos.” These developing embryos were found in significant proportions from day 17 and maintained high proportions on day 21 and later, until day 30 (**Figures 9B,C** and **10B**). Developing embryos were not found at earlier stages, during the first time points studied, when proembryos were abundant in the cultures (10–12 days; **Figure 10B**). By contrast, in AzaC-treated cultures, the progression of embryogenesis was inhibited and developing embryos were found in extremely low proportions at all the time intervals analyzed (**Figures 9E,F** and **10B**).

## Discussion

### DNA Hypomethylation by AzaC Induces Changes in the Chromatin Condensation Pattern and Promotes Microspore Reprogramming and Embryogenesis Initiation

*In vivo* exposure to 5-AzaC prevents the incorporation of methyl groups to DNA cytosines leading to DNA hypomethylation. Recently, we have shown that the microspore reprogramming to embryogenesis is accompanied by modifications in global DNA methylation which exhibits low levels after induction and early embryogenesis (Solís et al., 2012; El-Tantawy et al., 2014; Rodriguez-Sanz et al., 2014a). Therefore, with the aim of exploring whether epigenetic inhibitors could affect the DNA methylation dynamics during microspore embryogenesis, we studied the effects of the demethylating agent AzaC on the process and its potential application to improve microspore embryogenesis induction.

The present work was aimed to analyze the effects of the demethylating agent AzaC on microspore embryogenesis induction and progression, by comparing two different plant species, the monocot barley and the dicot rapeseed. These species are model systems for the process in which direct embryogenesis is induced, via different temperature stress treatments, in isolated microspores cultured in liquid media. The results of the short AzaC treatments demonstrated a positive effect of the drug on microspore embryogenesis induction, at the low concentration of 2.5 μM, increasing the percentage of microspore-derived proembryos formed, in the two systems.

AzaC has previously been tested as an additive in the culture medium of various *in vitro* systems of somatic embryogenesis and organogenesis, mainly through the culture of organs and tissue segments, with varying results. Most studies reported negative effects of the drug in the production of somatic embryos (Pedrali-Noy et al., 2001; Santos and Fevereiro, 2002; Yamamoto et al., 2005; Nic-Can et al., 2013; Teyssier et al., 2014); there are only a few examples in which AzaC promoted organogenesis or somatic embryogenesis (Li et al., 2001; Belchev et al., 2004; Tokuji et al., 2011; Fraga et al., 2012). In these previous studies, the range of concentration of AzaC has been very variable and high (from 10 to 200 μM). Therefore, a dose response effect with possible secondary effects and cell toxicity could occur in these *in vitro* systems, as previously reported (Juttermann et al., 1994; Teyssier et al., 2014). In addition, data on AzaC effects on early events of the process have not yet been analyzed. In the present work, lower concentrations of AzaC have been tested, 2.5, 5, and 10 μM, and their effects on cell death have been evaluated; the results of these analyses reveal that cultures with the lowest AzaC dose (2.5 μM) showed slightly lower proportions of dead cells than non-treated cultures, indicating that at this concentration the drug has no toxic effects on isolated microspore cultures. Therefore, 2.5 μM was the concentration selected for the treatments. Moreover, the quantification of global DNA methylation indicates that 2.5 μM AzaC significantly decreased the DNA methylation level of cells in microspore cultures of the two species studied, at precisely the same culture stage as when we detected significant increases in proembryo formation. These results indicate that, in rapeseed and barley, while the stress treatment induces microspore reprogramming and proliferation, concomitantly, AzaC-induced DNA hypomethylation promotes microspore embryogenesis initiation and formation of proembryos a few days after culture initiation.

Reprogramming and acquisition of cellular totipotency involve activation of numerous genes associated with the new developmental program and/or repression of genes of the original cell program. The way in which differentiating plant cells remodel their gene expression program during the acquisition of cell totipotency is a central question which involves large-scale chromatin reorganization (Tessadori et al., 2007). Changes in chromatin organization and variations in the level of global DNA methylation have been associated with several different *in vitro* plant regeneration processes (Loschiavo et al., 1989; Miguel and Marum, 2011). Also during microspore embryogenesis, remodeling of the chromatin organization patterns have been characterized in various species like pepper, tobacco, and rapeseed (Testillano et al., 2000, 2002, 2005; Bárány et al., 2005; Seguí-Simarro et al., 2011). In these previous studies, comparative analyses were performed between the gametophytic and the sporophytic pathways followed by the microspore, permitting the identification of defined nuclear changes that occurred when the microspore reprogrammed and switched to embryogenesis. These reports showed that the change of developmental program and the activation of proliferative activity (at the initiation of embryogenesis) affected the functional organization of the nuclear domains, which changed their architecture and functional state accordingly. Ultrastructural and *in situ* localization approaches revealed the pattern and functional states of chromatin and demonstrated the relation between the nuclear activity and the degree of chromatin condensation/decondensation. Regardless of the heterochromatin distribution pattern typical of each species, after microspore embryogenesis induction, the pattern of chromatin was less condensed in proembryos than in cells that follow the gametophytic development. Early microspore proembryos were characterized by a typical decondensed chromatin pattern, also found in proliferating cells of several plant species (Testillano et al., 2000, 2002, 2005; Bárány et al., 2005; Seguí-Simarro et al., 2011). *De novo* auxin biosynthesis and accumulation has been recently reported in early microspore embryogenesis, from the first divisions (Rodriguez-Sanz et al., 2015). This auxin accumulation has been related to the activation of proliferative activity in the reprogrammed microspore and early proembryo cells.

The results of the ultrastructural analysis of the chromatin condensation patterns together with the 5mdC immunofluorescence and immunogold assays presented here illustrate that AzaC-treatments not only decrease global DNA methylation levels but also modify the distribution pattern of the methylated DNA in the nucleus leading to more decondensed chromatin patterns in proembryo cells. In *B. napus*, the size and number of heterochromatin masses, enriched in 5mdC, diminished in proembryo cells treated with AzaC. Also in barley, the hypomethylating drug affected methylated DNA distribution and chromatin condensation patterns, which changed into more decondensed chromatin threads. In animals, cell totipotency and pluripotency have been associated with a global chromatin reorganization and decondensation leading to the so-called “open chromatin state” in which specific histone modifications and DNA hypomethylation, among other factors, have been shown to be involved. This open chromatin structure is required for the cell to maintain its totipotent state, ready for transcriptional activation (Shi et al., 2008; Gaspar-Maia et al., 2011; Gonzalez-Muñoz et al., 2014). In animals, after fertilization and the formation of the zygote (totipotent) chromatin is decondensed and acquires specific epigenetic marks (Burton and Torres-Padilla, 2010). High mobility of core histones, remodeling of constitutive heterochromatin marks, and acquisition of specific permissive histone modifications have been suggested as required features for the chromatin state compatible with cellular reprogramming (Burton and Torres-Padilla, 2010; Boskovic et al., 2014; Lu and Zhang, 2015). In plants, cellular reprogramming has been associated with nuclear changes including chromatin decondensation, reduction in heterochromatin and changes in DNA methylation and histone modifications landscapes (Solís et al., 2012; She et al., 2013; El-Tantawy et al., 2014; Rodriguez-Sanz et al., 2014b). In *Arabidopsis*, after fertilization, distinct chromatin patterns have been reported in the zygote (totipotent) and endosperm (Pillot et al., 2010), patterns that have been associated with differential epigenetic and transcription patterns in the zygote/embryo and endosperm (Pillot et al., 2010) and could underlay the totipotency acquisition in the zygote. By contrast, DNA hypermethylation, and repressive histone modifications has been associated with heterochromatization and cell differentiation in animal and plant systems (Lippman et al., 2004; Solís et al., 2012; El-Tantawy et al., 2014; Rodriguez-Sanz et al., 2014b).

Recently, it has been shown that the change of developmental program of the microspore toward embryogenesis is accompanied by modifications in global DNA methylation (Solís et al., 2012; El-Tantawy et al., 2014; Rodriguez-Sanz et al., 2014a) and changes in histone epigenetic modifications (Rodriguez-Sanz et al., 2014b). These facts indicate that an epigenetic reprogramming occurs after the induction of the microspore to a totipotent state and embryogenesis initiation. Recent work by our group with *B. napus* (Rodriguez-Sanz et al., 2014b) suggested the participation of the dimethylated histone H3K9me2, a repressive mark, and histone methyl transferases (HKMTs) in microspore embryo cell differentiation and heterochromatinization events, whereas the acetylated histones H3Ac and H4Ac, permissive marks, and histone acetyl transferases (HATs) were involved in transcriptional activation and totipotency during microspore reprogramming. In addition, the reported changes of the DNA methylation (Solís et al., 2012) that occur after microspore embryogenesis induction lead to low methylation levels in early embryo stages. DNA hypomethylation is associated with the change of developmental program and with the activation of cell proliferation at the beginning of embryogenesis, and this DNA hypomethylation appears to be related to a global change of gene expression (Solís et al., 2012). AzaC would facilitate/promote DNA hypomethylation and chromatin decondensation of cells stimulating reprogramming, totipotency acquisition, and early proembryo divisions and, therefore, increasing the efficiency of embryogenesis initiation. In mammalian cells, AzaC has been reported to induce expression of silenced genes, through demethylation of specific genome regions, and even to increase the expression of unmethylated genes by affecting histone methylation (Zheng et al., 2012). The DNA hypomethylation induced by AzaC could favor the deactivation of the gene expression program of the microspore to the pathway and the activation of a new gene expression program which promotes totipotency of a differentiating cell, the microspore, and the beginning of its active proliferation and cell cycle division.

*In vivo* exposure of *Allium cepa* root meristems to 5-AzaC (10^-6^M) stimulated the rate of nucleologenesis and shortened its cycle time (De-La-Torre et al., 1991; Mergudich et al., 1992). In AzaC-treated proliferating root cells, nucleoli on the hypomethylated NORs were larger, a sign of high transcriptional activity, as demonstrated by the increase of the rate of [^3^H]uridine incorporation in AzaC-treated root cells (Mergudich et al., 1992). The vacuolated microspore, the most responsive stage for embryogenesis induction, has been characterized by a high transcriptional activity which is reflected by a large nucleolus and a decondensed chromatin pattern (Testillano et al., 2000, 2005; Seguí-Simarro et al., 2011). The positive effect of AzaC on microspore embryogenesis induction could also be due in part to the activation of nucleolar activity and nucleologenesis rate which would promote cell cycle divisions of the reprogrammed microspore.

Furthermore, the results presented here show that the same effects of AzaC (DNA hypomethylation, chromatin decondensation and an increase in microspore embryogenesis induction rates) are found in the two species studied, a monocot and a dicot plant, suggesting common epigenetic mechanisms during microspore embryogenesis induction in both phylogenetic groups.

### DNA Methylation is Required for Microspore Embryo Differentiation and Long AzaC Treatment Prevents the Subsequent Embryo Development

In the present work, we have also analyzed the effects of the demethylating agent AzaC on the progression of microspore embryogenesis during subsequent developmental stages after the induction and the formation of proembryos. For this purpose, longer treatments of 2.5 μM AzaC were applied to microspore cultures. The results revealed that, in contrast with short AzaC treatments which promoted embryogenesis initiation and proembryo formation, longer treatments prevented subsequent embryogenesis progression. The proembryos formed in AzaC-treated cultures during the first days of treatment were also observed during the following days and, although their development had stopped, they did not show any aberrant morphology.

During development, in relation to differentiation processes, the pattern of DNA methylation in the genome changes as a result of a dynamic process involving both *de novo* DNA methylation and demethylation. As a consequence, differentiated cells acquire a stable and unique DNA methylation pattern that regulates tissue-specific gene transcription. The progress of the cellular differentiation has been related to a rapid increase in global DNA methylation levels in various plant developmental processes (Costa and Shaw, 2006, 2007; Malik et al., 2012). In mammals, heterochromatin increases dramatically during terminal cell differentiation and this has been linked to increased levels of DNA methylation (Politz et al., 2013). In *Arabidopsis*, embryos with loss-of-function mutations of the DNA methyltransferases MET1 and CMT3 (responsible of methylating DNA) develop improperly, indicating that DNA methylation is critical for plant embryogenesis (Xiao et al., 2006). Recent studies by our group have demonstrated the increase of global DNA methylation during microspore embryogenesis progression in rapeseed (Solís et al., 2012) and barley (El-Tantawy et al., 2014). This hypermethylation was associated with the heterochromatization that accompanies cell differentiation in advanced embryogenesis stages (Solís et al., 2012; El-Tantawy et al., 2014). In addition, the gene expression of the *MET1* DNA methyltransferase has been reported to increase during late stages of pollen maturation, tapetum developmental PCD, and differentiation of embryos originated from zygotes and microspores, in *B. napus* (Solís et al., 2012, 2014). This increase in *MET1* expression correlated with the increase in global DNA methylation and heterochromatization events (Solís et al., 2012, 2014). In the present work, the dynamics of DNA methylation has been altered by a demethylating agent, AzaC. The analysis of the effects of AzaC on the progression of microspore embryogenesis reported here showed that the drug clearly prevented embryo differentiation (hypermethylated stage), whereas AzaC promoted embryogenesis initiation (hypomethylated stage). The presence of the drug from the beginning until advanced stages blocked the process at the proembryo stage, which indicates that *de novo* DNA methylation is required for subsequent microspore embryo differentiation processes.

## Conclusion

Epigenetic inhibitors affecting DNA methylation, such as AzaC, provide a promising way for intervention through pharmacological assays to improve the efficiency of plant regeneration by stress-induced embryogenesis *in vitro* systems, as well as a convenient tool to investigate the role of DNA methylation dynamics in these processes. The results reported here demonstrated that AzaC increases microspore embryogenesis induction rates by inducing DNA hypomethylation and chromatin decondensation, at early stages. By contrast, subsequent embryo development is drastically affected by AzaC, suggesting that microspore-derived embryo differentiation requires *de novo* DNA methylation. The present study illustrates that low concentration and short duration of the AzaC treatment, at defined early stages, are critical points to achieve positive effects in terms of microspore embryogenesis efficiency, 2.5 μM AzaC for 4 days from culture initiation is a suitable treatment for promoting the induction of the process in isolated microspore cultures of two different species, rapeseed and barley. The results suggest common epigenetic mechanisms in both monocot and dicot plant systems and open the way to design new biotechnological strategies for improving doubled-haploid production in crop breeding programs.

## Conflict of Interest Statement

The authors declare that the research was conducted in the absence of any commercial or financial relationships that could be construed as a potential conflict of interest.
