# Corrigendum: 5-azacytidine promotes microspore embryogenesis initiation by decreasing global DNA methylation, but prevents subsequent embryo development in rapeseed and barley

**DOI:** 10.3389/fpls.2024.1514893

**Published:** 2024-11-05

**Authors:** María-Teresa Solís, Ahmed-Abdalla El-Tantawy, Vanesa Cano, María C. Risueño, Pilar S. Testillano

**Affiliations:** Pollen Biotechnology of Crop Plants Group, Biological Research Center (CIB) – Spanish National Research Council (CSIC), Madrid, Spain

**Keywords:** microspore culture, epigenetic inhibitors, demethylating agents, totipotency, microspore reprogramming, *Hordeum vulgare*, *Brassica napus*

In the published article, there was an error in the legend for **Figure 1** as published. The image corresponding to **Figure 1A** was re-used from a previous paper of the same authors without citation in the figure legend to the previous publication and the journal giving the permission of re-use. The corrected legend appears below.

**FIGURE 1:**
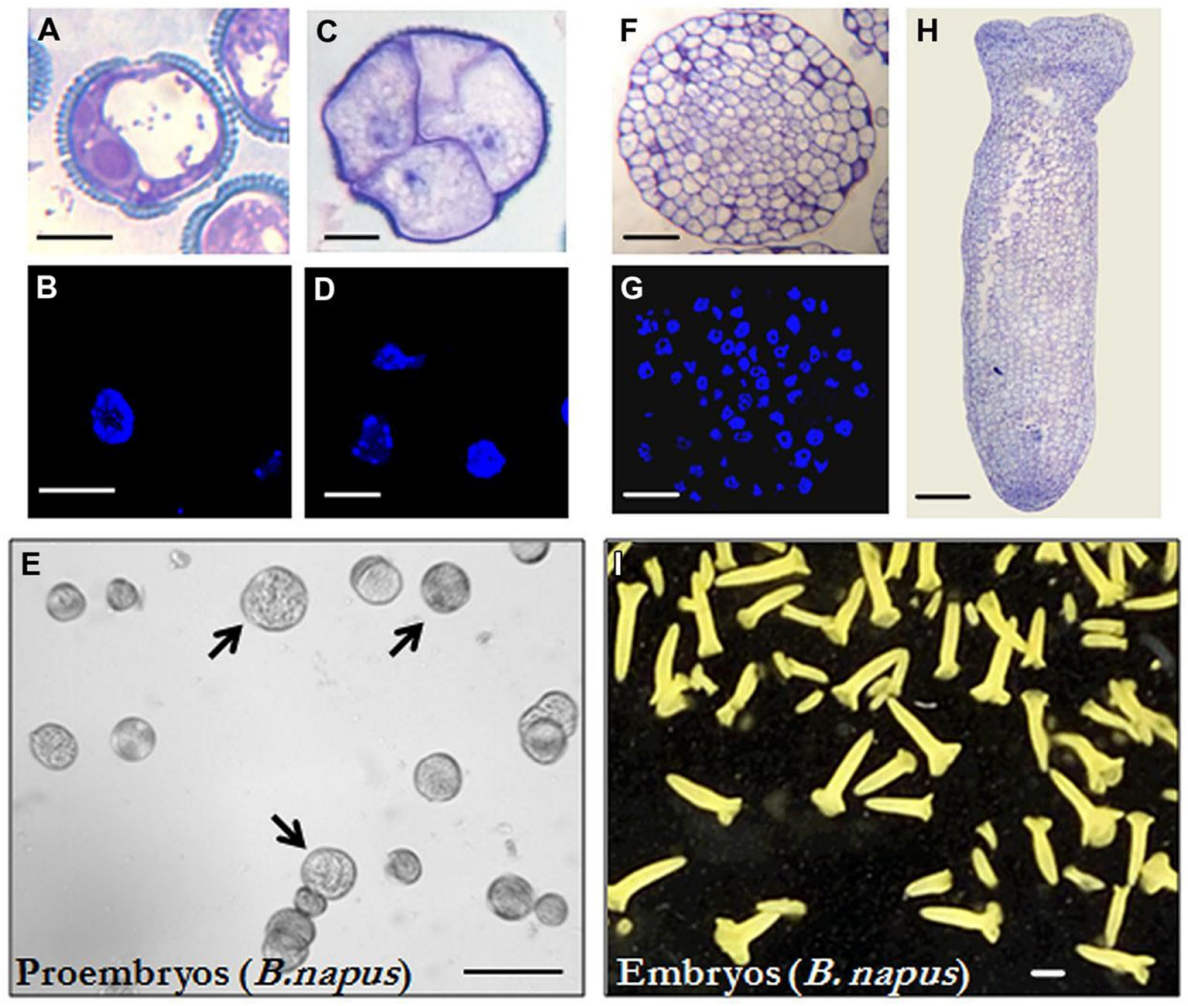
Microspore embryogenesis in *Brassica napus*. **(A, B)** Vacuolated microspores at the beginning of the culture. Figure 1A was reproduced from Figure 1A of Rodríguez-Sanz et al., 2014b (copyright ^©^ 2014 Karger Publishers, Basel, Switzerland). **(C, D)** Proembryos formed by four cells, still surrounded by the exine (the microspore wall). **(E)** *In vitro* culture at the proembryo formation stage (4 days), proembryos are pointed by arrows. **(F, G)** Globular embryos. **(H)** Torpedo embryo. **(I)** *In vitro* culture at the embryo production stage (30 days), most embryos show the typical morphology of cotyledonary embryos of the dicot embryogenesis pathway, some embryos at earlier developmental stages (heart and torpedo embryos) are also present. **(A, C, F, H)** Micrographs of toluidine blue-stained sections for general structure visualization. **(B, D, G)** DAPI staining for nuclei visualization (blue). **(E, I)** General views of cultures observed under the stereomicroscope. Bars represent, in **(A–D)** 10 μm, in **(E)** 250 μm, in **(F, G)** 50 μm, in **(H)** 100 μm, in **(I)** 1mm.

In the published article, there was no **Data Availability Statement**. Now, due to the time elapsed, the original data are not available. The relevant new created data of the article was contained within the publication.

The authors apologize for these errors and state that this does not change the scientific conclusions of the article in any way. The original article has been updated.

